# Incidence and outcomes of critical illness in Indigenous Peoples: a systematic review protocol

**DOI:** 10.1186/s13643-022-01948-x

**Published:** 2022-04-13

**Authors:** Samantha L. Bowker, Kienan Williams, Aireen Wingert, Jamie M. Boyd, Melissa L. Potestio, Michelle Gates, Erica Wright, Sean M. Bagshaw

**Affiliations:** 1 Critical Care Strategic Clinical Network™, Alberta Health Services, Edmonton, AB Canada; 2 Department of Critical Care Medicine, Faculty of Medicine and Dentistry, University of Alberta, 2-124 Clinical Science Building, 8440-112 Street NW, Edmonton, AB T6G2B7 Canada; 3 Indigenous Wellness Core™, Alberta Health Services, Calgary, AB Canada; 4 Alberta Research Centre for Health Evidence, University of Alberta, 4-486C Edmonton Clinic Health Academy, 11405 – 87 Avenue, Edmonton, AB T6G 1C9 Canada

**Keywords:** Indigenous People, Critical illness, Critical care, Health outcomes, Epidemiology

## Abstract

**Background:**

Indigenous Peoples experience health inequities across the continuum of health services. Improvements for Indigenous patients and their families during vulnerable experiences with the healthcare system may have a significant impact on the patient experience and outcomes. Improved understanding of the occurrence of critical illness in Indigenous Peoples and their use of critical care services, as a strategic priority, may aid in the development of initiatives for improving health equity. A global focus was selected to learn from Indigenous populations’ experiences with critical care, as the understanding of critical illness among Indigenous Peoples in Canada is not well understood. This protocol outlines a systematic review focused on describing the incidence of critical illness and utilization of critical care services among Indigenous Peoples.

**Methods:**

Ovid MEDLINE/PubMed, Ovid EMBASE, Google Scholar, and Cochrane Central Register of Controlled Trials will be searched. Relevant Canadian sites for gray literature (National Collaborating Centre for Indigenous Health, First Nations Health Authority, Canadian Institutes of Health Research Institute of Indigenous Peoples’ Health, National Association of Friendship Centres, the Alberta First Nations Information Governance Centre, Métis Nation of Alberta) will also be searched. We will include studies of adults (≥18 years) either without critical illness (i.e., general population) or with critical illness (i.e., admitted to an intensive care unit (ICU)). The exposure of interest will be Indigenous identity. Primary outcome measures are ICU admission and ICU mortality. Because heterogeneity in populations, comparisons, and outcome measures is anticipated, it is likely that the findings will be summarized using a narrative synthesis. A meta-analysis will be performed if there is sufficient evidence on one or more outcomes of interest.

**Discussion:**

This systematic review will provide a better understanding of the epidemiology, risk factors, and outcomes of critical illness and utilization of critical care services among Indigenous Peoples. The knowledge generated will be applied to a broader program of work designed to create ethical space to co-design, implement, and evaluate a culturally competent, safe, and innovative model for critical care services for Indigenous People.

**Systematic review registration:**

PROSPERO CRD42021254661

**Supplementary Information:**

The online version contains supplementary material available at 10.1186/s13643-022-01948-x.

## Background

Health inequities for Indigenous People arise from the intergenerational impact of colonization and need to be contextualized within the historical, political, social, and economic conditions that have influenced Indigenous health [1–3]. These inequities span across the healthcare continuum from birth to death, with mortality rates for Indigenous Peoples being twice that of non-Indigenous populations (e.g., Indigenous: 742/100,000 per year vs non-Indigenous: 359/100,000 per year) [4]. Prioritizing equity requires that we build a healthcare system that meets the unique needs of specific populations and works to help identify, prioritize, and overcome barriers to ensuring the provision of high-quality services in pursuit of optimal health outcomes. For Indigenous Peoples, this means building a healthcare system that recognizes and respects Indigenous leadership over their own health matters, makes space for community voices and priorities, and integrates cultural competencies in health service practice [5, 6]. It requires building and nurturing meaningful and sustained relationships with Indigenous communities. It further necessitates recognizing the wisdom of Indigenous wellness practices and their expertise in their own lives.

A strong culturally competent healthcare system, inclusive of critical care, is one key action to reducing health inequities experienced by Indigenous Peoples [7–9]. The sickest patients in the hospital are cared for in intensive care units (ICUs), where enhanced monitoring and multi-organ life-sustaining therapies are provided [10, 11]. Critical illness among Indigenous Peoples and the use of critical care services are not well understood, particularly within Canada [12, 13]. To be positioned to work within an ethical space to reduce health inequities and improve quality of care, we must understand the incidence of critical illness as well as the correlates and use of critical care services among Indigenous Peoples in Canada [5]. Accordingly, we will perform a systematic review to better understand access and use of critical care services, including the incidence of critical illness, the risk factors associated with critical illness, and the short- and long-term outcomes of critical illness among Indigenous Peoples, compared (when possible) to non-Indigenous people.

## Methods

### Study design

We will perform a systematic review guided by standard evidence synthesis methodology [11]. The study protocol is reported according to the Preferred Reporting Items in Systematic Reviews and Meta-Analyses (PRISMA) statement [14].

### Study registration

The study protocol is registered with PROSPERO Register of Systematic Reviews (registration number: CRD42021254661; June 12, 2021).

### Eligibility criteria

Table 1 shows the full eligibility criteria for inclusion of studies in the systematic review. The population of interest will be adults (≥18 years) either without critical illness (i.e., general population) or with critical illness (i.e., admitted to an intensive care unit (ICU)). The exposure of interest will be Indigenous identity. Within Indigenous populations, we will ascertain the following factors: age, sex/gender, setting, presence of comorbidities, acuity of illness (e.g., APACHE II, SOFA), primary diagnosis (e.g., trauma, sepsis, surgery), and others as reported by study authors.

**Table 1:** Eligibility criteria for inclusion of studies in the review

	Inclusion and exclusion criteria
**Population**	**Include:** Adults (≥18 years) without critical illness in the general population; hospitalized for any reason; or with critical illness (i.e., admitted to ICU). Studies that include children will be considered if the population mean age – 1 standard deviation is ≥18 years. **Exclude:** Children and adolescents and studies where the mean age – 1 standard deviation is <18 years.
**Exposures**	**Include:** Indigenous identity as the primary focus; within Indigenous populations, we will consider risk factors including age, sex/gender, presence of comorbidities, acuity of illness (e.g., APACHE II, SOFA), primary diagnosis (e.g., trauma, sepsis, surgery), setting, and others as reported by the included studies. **Exclude:** Studies focused exclusively on non-Indigenous populations, studies with mixed populations where data specific to Indigenous Peoples cannot be isolated or the sample of the Indigenous population is less than 5% of the total study population; studies focused on a population with an identified condition and/or procedure, and where Indigenous identity is examined as a risk factor/covariate (i.e., Indigenous is not the primary focus)
**Comparators**	**Include:** - For incidence of ICU admission, studies require a comparator group with non-Indigenous identity (“general population” and “hospitalized”). We will include studies without a comparator group if they investigate risk factors for ICU admission within the Indigenous population. - For ICU-admitted populations, we will include studies with or without a non-Indigenous comparison group. **Exclude:** Comparisons between different Indigenous groups.
**Outcomes**	**Include:** Incidence of critical care admission; associations between risk factors (age, sex, acuity), and critical care access; and critical care outcomes. **Primary:** - ICU admission^a^ - ICU mortality **Secondary:** - ICU length of stay, ICU re-admission, mechanical ventilation, duration of mechanical ventilation, tracheostomy, vasoactive support, duration of vasoactive support, acute dialysis, duration of acute dialysis, advanced life support, total hospital length of stay, hospital mortality, rehospitalization, quality of life measured on any scale
**Study designs**	**Include:** Prospective and retrospective cohort studies, case series with aggregate data from at least 100 participants, clinical trials (e.g., usual care or placebo arm), analytic findings from gray literature reports **Exclude:** Evidence syntheses, descriptive and cross-sectional studies, case reports, and other reports that do not present any original data (e.g., commentaries, editorials, opinion pieces)
**Settings**	Any country
**Time period**	For mortality: in ICU, in hospital, 28 days, 1 year, >1 year For rehospitalization: 0–6 months, 6–12 months, any longer length of follow-up For quality of life: measured at any point in time For other outcomes, any time point or length of follow-up is eligible.
**Language**	**Include:** English **Exclude:** Any language other than English
**Date of publication**	Any

*ICU* Intensive care unit

^a^ICU is defined as a hospital unit designed to provide invasive therapies (e.g., mechanical ventilation) in order to sustain human life, with admission defined as support in a hospital location designated as ICU for ≥24 h

For outcomes of interest, we will include incidence of critical care admission, associations (e.g., odds, risk, or hazard ratio) between potential risk factors and critical care admission, and critical care outcomes. We will prioritize the following risk factors: age, sex, and acuity. Primary outcome measures were pre-defined as ICU admission and ICU mortality; secondary outcomes are listed in Table 1. For the primary outcome of ICU admission, we will only include studies with a non-Indigenous “general population” or “hospitalized” comparator. If studies in the general or hospitalized population do not have a non-Indigenous comparison group, we will only include studies on the Indigenous population that also have information on the following risk factors within the Indigenous population: age, sex, and acuity. For studies that look at critical care outcomes (such as ICU mortality), we will include studies with and without a non-Indigenous comparator.

We will include the following study designs: observational cohort studies (both retrospective and prospective), case series reporting aggregate data on at least 100 patients, arms of clinical trials (e.g., usual care, or placebo arm), and analytical data from gray literature reports. Descriptive studies, cross-sectional studies, case reports, and articles that do not present original data (e.g., opinion pieces, editorials, commentaries) will be excluded. We will include studies from all countries that are published in English.

For this review, critical illness is defined by the complexity of illness, severity of organ dysfunction, and risk of mortality, requiring complex therapies and treatments (e.g., mechanical ventilation) to sustain human life that requires admission to an intensive care unit [10, 11]. Critical care access and/or utilization is defined as admission to an ICU or support in a hospital location designated as an ICU for ≥24 h. The definition of “Indigenous identity” varies based on country and academic discussions on culturally appropriate terminology [15]. For studies that present both Indigenous and non-Indigenous patient data, data will only be extracted if Indigenous patient data can be isolated. Studies that report on both adult and youth (<18 years) patient data will be included if data specific to adults can be isolated, or if the mean age -1 standard deviation of the study population is ≤ 18 years.

### Search strategy and information sources

The search strategy will be developed in consultation with the Alberta Research Centre for Health Evidence (ARCHE) at the University of Alberta, first with an information specialist and then peer-reviewed by a second research librarian [16]. The search strategy will include two groups of terms (keywords with similar characteristics): “Indigenous Peoples” and “critical care” (see Additional file 1 for the search strategies implemented in each database). Keywords for each respective group will be combined using the Boolean operator “OR.” We will then combine the groups together using the Boolean operator “AND.” We will systematically search the following electronic databases from inception to present, restricted by the English language: Ovid MEDLINE/PubMed, Ovid EMBASE, Google Scholar, and Cochrane Central Register of Controlled Trials. We will additionally search relevant Canadian websites for gray literature (National Collaborating Centre for Indigenous Health, First Nations Health Authority, Canadian Institutes of Health Research Institute of Indigenous Peoples’ Health, National Association of Friendship Centres, the Alberta First Nations Information Governance Centre, Métis Nation of Alberta). Hand-searching bibliographies of included studies and relevant systematic reviews will be used to locate additional citations. EndNote (Thompson Reuters, Philadelphia, PA, USA) will be the reference software used for the management of citations. See Additional file 1 for the full search strategy and terms.

### Study screening for inclusion

Both abstract title (level 1) and full text (level 2) reviews will have pre-determined eligibility criteria that will be applied by reviewers for each respective stage. Level 1 criteria are broader than level 2 to ensure all potentially relevant studies are captured. Reviewers will pilot the inclusion criteria on randomly selected articles (5% of total citations) prior to each stage, with additional pilot rounds as needed to ensure a high level of agreement. Two reviewers will independently, and in duplicate, review the titles and abstracts of the retrieved citations. Full-text articles will be obtained for all titles and abstracts identified by one or both reviewers as potentially relevant. Two reviewers will then independently, and in duplicate, identify full-text articles meeting the inclusion criteria. Consensus will be used to resolve eligibility disagreements between reviewers and a third reviewer with clinical expertise will be consulted if consensus cannot be reached. A PRISMA flow diagram will be used to illustrate the flow of citations included and excluded in the systematic review [14].

### Data extraction

Following a pilot round to ensure consistency, two reviewers will independently extract data into a standardized electronic form. Data will be extracted on (1) study characteristics (e.g., design, country, setting, language, number of ICUs, type of ICU, data source, length of follow-up, funding source), (2) study purpose and eligibility criteria, (3) patient characteristics (e.g., Indigenous population, sample size, age, sex/gender, illness severity, comorbidities), (4) information related to risk of bias, (5) comparators of interest, and (6) information related to study outcomes (e.g., definitions, ascertainment method, time points, and relevant findings). It is anticipated there will be significant heterogeneity between included studies, both in reported outcomes and units of measure. As such, study data will be extracted as reported within the article using the units of measure reported. A second reviewer will verify the extracted data to correct errors or omissions. Discrepancies will be resolved between the reviewers through consensus and referred to a third reviewer, if required. When important study data are missing or unclear, we will contact study authors for clarification twice, 2 weeks apart.

### Methodological quality assessment

Following a pilot round, two reviewers will independently assess the methodological quality of the included studies using the Newcastle-Ottawa Scale (NOS) across the following domains: selection of the exposed and unexposed cohorts, comparability of the cohorts, and outcome ascertainment [17]. For comparability, we will require that groups are comparable, or controlled for age, sex/gender, and level of acuity, at minimum. Scores will be summed across the domains for a maximum score of 9 for each study. We will rate studies as poor quality (<4/9), moderate quality (4–6/9), and high quality (>6/9). Disagreement in ratings between the two reviewers will be resolved by discussion or by consulting a third reviewer, if needed.

### Data synthesis

It is anticipated that there will be heterogeneity in populations, comparisons, and outcome measures. Therefore, it is likely that the findings will be summarized using a narrative synthesis approach for systematic reviews [18]. As such, data will be presented in tables and text to summarize the study characteristics, findings, and limitations. Descriptive statistics will be presented on the study and patient characteristics. Studies will be classified first according to the methodological quality for the pre-specified primary outcomes of ICU admission and ICU mortality. Dichotomous data will be presented as counts and percentages or risk ratio/odds ratio and 95% confidence interval, depending on available data reported. Continuous data will be reported as mean (standard deviation) or median (interquartile range). When possible, we will standardize the outcome measures reported across studies for ease of interpretation.

If there is adequate clinical and methodological homogeneity for an outcome-comparison of interest, we will perform a meta-analysis and pool the findings statistically using Review Manager version 5.4 (The Nordic Cochrane Centre, the Cochrane Collaboration, Copenhagen, Denmark). Statistical analyses will be performed using Stata SE version 13.1 (Stata Corp, LP, College Station, TX). Heterogeneity will be quantified using the *I*^2^ statistic and will be explored using between-study subgroup analyses (e.g., by age, sex/gender, level of acuity). If applicable, we will perform sensitivity analyses to understand the effects of variably defined exposures and/or outcomes and study quality. For meta-analyses including at least eight studies of varying size, we will test for small study bias by interpreting funnel plots and statistically using Egger’s test [19].

### Confidence in cumulative evidence

Two reviewers will independently assess the certainty of evidence for the primary outcomes using the Grading of Recommendations, Assessment, Development, and Evaluation (GRADE) approach [20–22]. As the best evidence for prognostic factors originates from observational studies [23], evidence from these will start at high certainty and be rated down for concerns about the risk of bias (methodological quality), inconsistency, indirectness, imprecision, and other concerns. Inconsistencies between reviewers will be resolved by discussion or the involvement of a third reviewer, if needed.

### Project team and indigenous engagement

This project aims to bring together Western and Indigenous research approaches and understandings [6, 24]. The project is a partnership between the *Indigenous Wellness Core™ (IWC™)* and the *Critical Care Strategic Clinical Network™ (CC SCN™)* of Alberta Health Services [25, 26]. The current project leads include Samantha Bowker, a non-Indigenous epidemiologist, health researcher and Assistant Scientific Director of the *CC SCN™*; Kienan Williams, an Anishnawbe health researcher, member of the Sandy Lake First Nation, and Program Lead, Innovation and Research for the *IWC*™; and Sean Bagshaw, a non-Indigenous clinician-scientist, Canada Research Chair in *Critical Care Outcomes and Systems Evaluation*, and Scientific Director of *CC SCN™*. The project team strongly believes any work without Indigenous Peoples’ voice risks perpetuating existing knowledge and care gaps. As such, during synthesis of evidence and drafting of the final manuscript prior to publication, the project leads will invite members of the Indigenous community to engage in discussion of the findings, provide context for interpretation based on Indigenous Ways of Knowing, and ensure the perspectives of the Indigenous Peoples have been appropriately integrated [24].

## Discussion

Health inequities exist for Indigenous People that are a consequence of colonization and racism. Indigenous Peoples in Canada have a disproportionately high prevalence of several chronic diseases, including diabetes mellitus, hypertension, and cardiovascular disease [8, 27–29]. For example, Indigenous Peoples live 10 fewer years of life compared to the general population [4, 29]. Social determinants of health (such as a socioeconomic status, living in remote areas, and jurisdictional barriers in access to health care) are significant contributors to health inequities among Indigenous Peoples [30]. When a person experiences a life-threatening injury or illness, they should not wonder if their children will be apprehended by child services, or if the last words they hear will be racist comments from clinicians [26, 31, 32]. These are very real concerns for Indigenous People.

Despite the well-recognized health inequities of Indigenous Peoples, very little is known about the experience and outcomes of Indigenous People in critical care. An expected limitation are publications where Indigenous People are the object of study by non-Indigenous individuals, with implicit biases impacting the analysis. This review will privilege Indigenous voices and perspectives on accessing critical care services, while acknowledging the publicly available data may be limited in this regard.

This review will serve as a foundational step towards understanding the development of critical illness and the use of critical care services for Indigenous Peoples. The knowledge generated will be applied to a broader program of work designed to create ethical space to co-design, implement, and evaluate a culturally competent, safe, and innovative model for critical care services for Indigenous People [5, 6].

Any major amendments to this systematic review protocol following publication will be decided by consensus among the authors and included in the final manuscript.

## Supplementary Information

**Additional file 1.** Full search strategy and search terms.

## Data Availability

Not applicable.

## Abbreviations

ICU: Intensive care unit
PRISMA: Preferred Reporting Items in Systematic Reviews and Meta-Analyses
APACHE: Acute Physiology and Chronic Health Evaluation
SOFA: Sequential Organ Failure Assessment
ARCHE: Alberta Research Centre for Health Evidence
NOS: Newcastle Ottawa Scale
GRADE: Grading of Recommendations, Assessment, Development, and Evaluation
