# Supplementary material for: Incidence and outcomes of critical illness in Indigenous Peoples: a systematic review protocol
Source: Syst Rev. 2022 Apr 13;11:65. doi: 10.1186/s13643-022-01948-x (PMC9006439; doi:10.1186/s13643-022-01948-x)
Supplement: Supplementary file 1 — Additional file 1. Full search strategy and search terms. [file 13643_2022_1948_MOESM1_ESM.docx]

**Additional file 1.** Full search strategy and search terms

Database: Ovid MEDLINE(R) ALL <1946 to May 03, 2021>

Search Strategy: Indigenous-Critical Care MEDLINE

--------------------------------------------------------------------------------

1 exp American Native Continental Ancestry Group/ (21889)

2 exp Indigenous Peoples/ (4819)

3 Health Services, Indigenous/ (3463)

4 Northern Territory/ (1501)

5 Arctic Regions/ (6798)

6 Nunavut/ (345)

7 Oceanic Ancestry Group/ (10744)

8 aborigin*.ti,ab,kf. (10374)

9 ((American or Northamerican) adj1 Indian*).ti,ab,kf. (7215)

10 amerindian*.ti,ab,kf. (2034)

11 (First adj1 (Nation or Nations or People)).ti,ab,kf. (2197)

12 indigenous*.ti,ab,kf. (35424)

13 (eskimo* or inuit*).ti,ab,kf. (3375)

14 maori*.ti,ab,kf. (3669)

15 metis*.ti,ab,kf. (445)

16 (Native* adj1 (American* or Alaska* or Australia* or Canadian* or Northamerican* or Hawaiian*)).ti,ab,kf. (10628)

17 Nunavik*.ti,ab,kf. (250)

18 Nunavut*.ti,ab,kf. (567)

19 Torres Strait Islander*.ti,ab,kf. (1828)

20 or/1-19 (84520) [Indigenous Population Terms]

21 Critical Care/ (54606)

22 intensive care units/ (58790)

23 ((intensive or critical) adj (care or medicine)).ti,ab,kf. (181739)

24 (ICU or ICUs).ti,ab,kf. (66609)

25 intensivist*.ti,ab,kf. (3826)

26 Critical Illness/ (31668)

27 critical* ill*.ti,ab,kf. (55820)

28 Multiple Organ Failure/ (11272)

29 (multi* organ adj (disfunction* or dis function* or dysfunction* or dys function* or failure*)).ti,ab,kf. (15806)

30 (multi* system adj (disfunction* or dis function* or dysfunction* or dys function* or failure*)).ti,ab,kf. (292)

31 or/21-30 (279966) [Critical Care Terms]

32 20 and 31 (342)

33 limit 32 to english language (334)

***************************

Database: Embase <1974 to 2021 May 03>

Search Strategy: Indigenous-Critical Care Embase

--------------------------------------------------------------------------------

1 exp indigenous people/ (28355)

2 indigenous health care/ (962)

3 northern territory/ (462)

4 Arctic/ (8060)

5 nunavut/ (179)

6 exp oceanic ancestry group/ (7545)

7 exp eskimo-aleut people/ (2927)

8 exp malayo-polynesian people/ (4433)

9 mestizo/ or metis/ (810)

10 aborigin*.ti,ab,kw. (12957)

11 ((american or northamerican) adj1 Indian*).ti,ab,kw. (8740)

12 amerindian*.ti,ab,kw. (2592)

13 (First adj1 (Nation or Nations or People)).ti,ab,kw. (2917)

14 indigenous*.ti,ab,kw. (42766)

15 (eskimo* or inuit*).ti,ab,kw. (3412)

16 maori*.ti,ab,kw. (4579)

17 metis*.ti,ab,kw. (598)

18 (Native* adj1 (American* or Alaska* or Australia* or Canadian* or Northamerican*)).ti,ab,kw. (12718)

19 Nunavik*.ti,ab,kw. (277)

20 Nunavut*.ti,ab,kw. (627)

21 Torres Strait Islander*.ti,ab,kw. (2401)

22 or/1-21 (100577)

23 intensive care/ or intensive care nursing/ (132971)

24 intensive care unit/ (171573)

25 ((intensive or critical) adj (care or medicine)).ti,ab,kw. (275595)

26 (ICU or ICUs).ti,ab,kw. (135255)

27 intensivist*.ti,ab,kw. (7113)

28 critical illness/ (31703)

29 critical* ill*.ti,ab,kw. (85817)

30 multiple organ failure/ (41983)

31 (multi* organ adj (disfunction* or dis function* or dysfunction* or dys function* or failure*)).ti,ab,kw. (25883)

32 (multi* system adj (disfunction* or dis function* or dysfunction* or dys function* or failure*)).ti,ab,kw. (454)

33 or/23-32 (498333)

34 22 and 33 (750)

35 limit 34 to conference abstracts (272)

36 34 not 35 (478)

37 limit 36 to english language (465)

***************************

Cochrane Library on Wiley

Date Run: 05/05/2021 18:52:15

ID Search Hits

#1 [mh "American Native Continental Ancestry Group"] 296

#2 [mh ^"Health Services, Indigenous"] 42

#3 [mh ^"Medically Underserved Area"] 123

#4 [mh ^"Northern Territory"] 22

#5 [mh ^Nunavut] 2

#6 [mh ^"Oceanic Ancestry Group"] 173

#7 [mh "Indigenous Peoples"] 49

#8 aborigin*:ti,ab,kw 339

#9 ((American or Northamerican) near/1 Indian*):ti,ab,kw 567

#10 Eskimo*:ti,ab,kw 34

#11 (First near/1 (Nation or Nations or People*)):ti,ab,kw 187

#12 Indigenous*:ti,ab,kw 925

#13 Inuit*:ti,ab,kw 61

#14 Maori*:ti,ab,kw 237

#15 Metis*:ti,ab,kw 40

#16 (Native* near/1 (American* or Alaska* or Australia* or Canadian* or Northamerican* or Hawaiian*)):ti,ab,kw 488

#17 Nunavik*:ti,ab,kw 1

#18 Nunavut*:ti,ab,kw 6

#19 Torres Strait Islander*:ti,ab,kw 87

#20 {or #1-#19} 2512

#21 [mh ^"Critical Care"] 1762

#22 [mh ^"intensive care units"] 2369

#23 ((intensive or critical) near/1 (care or medicine)):ti,ab,kw 25821

#24 (ICU or ICUs):ti,ab,kw 13854

#25 intensivist*:ti,ab,kw 307

#26 [mh "critical illness"] 2353

#27 critical* ill*:ti,ab,kw 11231

#28 [mh "Multiple Organ Failure"] 417

#29 (multi* organ near/1 (disfunction* or dis function* or dysfunction* or dys function* or failure*)):ti,ab,kw 3587

#30 (multi* system near/1 (disfunction* or dis function* or dysfunction* or dys function* or failure*)):ti,ab,kw 540

#31 {or #21-#30} 39473

#32 #20 AND #31 in Trials 34
